# The Influence of Tobacco Smoking on the Relationship between Pressure and Flow in the Middle Cerebral Artery in Humans

**DOI:** 10.1371/journal.pone.0072624

**Published:** 2013-08-15

**Authors:** Karen C. Peebles, Helen Horsman, Yu-Chieh Tzeng

**Affiliations:** 1 Cardiovascular Systems Laboratory, University of Otago, Wellington, New Zealand; 2 Centre for Translational Physiology, University of Otago, Wellington, New Zealand; 3 Department of Physiology, University of Otago, Dunedin, New Zealand; Harbin Medical University, China

## Abstract

**Background:**

Cigarette smoking is associated with an increased risk of stroke but the mechanism is unclear. The study examined whether acute and chronic cigarette smoking alters the dynamic relationship between blood pressure and cerebral blood flow. We hypothesised that acute and chronic smoking would result in a cerebral circulation that was less capable of buffering against dynamic fluctuations in blood pressure. Further, these changes would be accompanied by a reduction in baroreflex sensitivity, which is reduced after smoking (acute smoking).

**Methods:**

We recruited 17 non-smokers and 15 habitual smokers (13 ± 5 pack years). Continuous measurements of mean cerebral blood flow velocity (transcranial Doppler ultrasound), blood pressure (finger photoplethysmography) and heart rate enabled transfer function analysis of the dynamic relationship between pressure and flow (gain, normalised gain, phase and coherence) and baroreflex sensitivity during supine rest before and after smoking a single cigarette (acute smoking).

**Results:**

There were no between-group differences in gain, phase or coherence before acute smoking. However, both groups showed a reduction in gain and coherence, associated with a reduction in baroreflex sensitivity, and increase in phase after acute smoking.

**Conclusions:**

Contrary to our hypothesis, these findings suggest that in the face of a reduction in baroreflex sensitivity acute smoking may potentially improve the ability of the cerebral circulation to buffer against changes in blood pressure. However, chronic smoking did not alter the dynamic relationship between blood pressure and cerebral blood flow velocity. These results have implications on understanding mechanisms for attenuating stroke risk.

## Introduction

Although cigarette smoking increases stroke risk in a dose-dependent manner [1,2] the pathogenic mechanisms underlying this risk association is poorly understood. Impaired baroreflex sensitivity (BRS) and consequent increases in blood pressure (BP) and BP variability [3,4] has been implicated in the development of stroke in smokers. However, given the cerebral circulation possesses adaptive mechanisms such as cerebral autoregulation (CA) that can accommodate BP alterations, the elevation of stroke risk associated with smoking may be due to local impairment of cerebrovascular function.

Most studies that have examined the effect of smoking on the cerebral circulation have focused on its effects on resting cerebral blood flow (CBF) or cerebral vascular reactivity to hypercapnia. Regarding the former, reports are mixed as to whether smoking increases [5] maintains [6] or decreases [7] CBF most likely due to methodological differences (e.g. smoking dose and time of measurement). Regarding the latter, acute smoking has been shown to reduce the cerebral vasodilatory response to hypercapnia [8,9]. Studies to date have not examined whether acute and chronic smoking impairs the cerebrovascular response to beat-to-beat alterations in BP across a range of time scales in the frequency domain.

Transfer function analysis (TFA) is a way of examining the relationship between BP (input) and CBF (output) in the frequency domain [10,11]. TFA between BP and CBF velocity (a surrogate measure of CBF) yields estimates of gain (the amplitudinal relationship between input and output), phase (temporal relationship between input and output) and coherence (linearity between input and output). Further analysis in the frequency domain, affords the opportunity to examine these metrics within discrete frequency bands, in particular the low frequency band (0.07-0.2 Hz) in which cerebral regulatory mechanisms are believed to be operant [12]. TFA is a useful analytical tool for analysing cerebral vascular regulation as, for example, previous studies have demonstrated that patients with cerebrovascular pathology exhibit changes in TFA metrics [13,14].

Therefore, the purpose of this study was to examine the impact of acute and chronic cigarette smoking on the dynamic relationship between BP and CBF using TFA. We hypothesised that smoking would render the cerebral circulation more vulnerable to systemic BP alterations, most likely in a dose-dependent manner and in combination with an impairment in baroreflex function.

## Methods

### Ethics and participant cohorts

Participants (18 healthy non-smokers and 15 habitual smokers) took part in this study having provided written informed consent. Procedures were approved by the New Zealand Central Regional Ethics Committee and confirmed to the guidelines set by the *Declaration of Helsinki*.

Participants were screened for medical disorders and their smoking status was recorded in pack years. Exclusion factors included a history of respiratory and cardio/cerebro-vascular disease and any medications known to alter autonomic function (e.g. β–blockers). Premenopausal female participants were not pregnant and were studied in the early follicular phase of their menstrual cycle.

Studies were conducted in a quiet, temperature-controlled laboratory (~22° C). Participants arrived at the laboratory at ~8.00 am after a light breakfast and having refrained from caffeine, alcohol and strenuous exercise for 12 h before testing. Habitual smokers also refrained from smoking for at least 6 h prior to experimentation; blood nicotine levels return to baseline levels within 3 h after smoking a cigarette [15].

### Instrumentation

Following cannulation of the antecubital vein, participants were instrumented as follows. Finger photoplethysmography (Finometer, TPD Biomedical Instrumentation) was used to measure arterial BP, and was verified at regular intervals using oscillometric BP measurements (Seinex Electronics Ltd, UK). Electrocardiography (ECG, ADInstruments, Colorado Springs, CO) was used record heart rate (HR). Pulsed-wave transcranial Doppler ultrasound (2 MHz probe, Spencer Technologies, Seattle) was used to measure CBF velocity at the M1 segment of the middle cerebral artery (MCA) [16]. A gas analyser (model ML206, ADInstruments, Colorado Springs, CO) was used to measure the fraction of end-tidal CO_2_ during nasal expiration. These were sampled continuously at 1 kHz using an analog-digital converter (Powerlab/16SP ML795; ADInstruments) and stored for off-line analysis.

### Protocol

After acclimatising to the equipment, a baseline blood sample (~5 ml) was drawn into Lithium Heparin-coated vacutainers. Subsequently, 10 min of supine resting data was collected before (pre-smoking baseline) participants smoked a commercial cigarette containing 1 mg nicotine (termed acute smoking). To standardise the procedure, participants inhaled for ~7 s before exhaling and inhalations were performed at ~15 s intervals. Then a post-smoking blood sample was drawn before another 10 min of supine resting data (post-smoking baseline) was collected. A 10-point visual analogue scale (VAS) was used to assess the participant’s symptoms of unwellness (e.g. dizziness, nasusea, headache, where 0 = no symptoms and 10 = severe symptoms) just before taking the two blood samples. Justification for studying the systemic and cerebral consequences of smoking within 10 min of completing the cigarette was based on data indicating that blood nicotine concentrations in the systemic circulation peak ~10 min after smoking a single cigarette [15] and nicotine reaches the brain and crosses the blood-barrier 10-20 s after inhalation [17].

### Data analysis

Haemodynamic and respiratory data were analysed using custom-written software in Lab View 8.2 (National Instruments, Austin, TX). From the recorded arterial waveform we determined beat-to-beat values for systolic (SAP), diastolic (DAP) and mean arterial pressure (MAP). From the CBF velocity waveform we calculated mean cerebral blood flow velocity (MCAv_mean_). Cerebrovascular conductance index (CVCi) was calculated as the MCAv_mean_ divided by the MAP. HR was calculated from the R-R interval of the ECG. The partial pressure of end-tidal CO_2_ (P_ET_CO_2_) was calculated by the product of the fraction of end-tidal CO_2_ and ambient barometric pressure. These data were averaged over the 10 min pre- and post-smoking collection periods to provide their respective values.

### Transfer function analysis

Before TFA the recorded ECG, BP and mean MCAv signals were checked for artifacts, and erroneously detected or missed ‘R’ waves were corrected by linear interpolation. Beat-to-beat R-R interval, SAP, MAP and MCAv signals were then resampled at 4 Hz and divided into 5 successive windows which overlapped by 50% before being passed through a Hanning window and fast-Fourier transformed [18].

In this study we analysed MAP power (input), MCAv power (output), gain (in absolute units, and also in normalised units derived as the percentage changes in MCAv [beat-to-beat values divided by their mean values] in relation to the changes in MAP), phase angle, and coherance, in the very low frequency (VLF, 0.02-0.07 Hz), low frequency (LF, 0.07-0.20 Hz) and high frequency (HF, 0.2-0.35 Hz) ranges. Typically, increases in low frequency gain and coherance (i.e. gain_LF_ and coherance_LF_, respectively) and decreases in low frequency phase (i.e. phase_LF_) are interpreted as reduced dynamic cerebral autoregulation (CA) [18] although changes in intrinsic vascular properties can also modulate dynamic pressure-flow relationships [19]. Spontaneous BRS was quantified as the linear transfer function between SAP (input) and R-R interval (output) within the low frequency range (LF, 0.04-0.15 Hz) where coherence is 0.5 [20].

### Blood analysis

Immediately after collection, blood samples were centrifuged and the plasma frozen for later batch analysis. Nicotine and cotinine levels were analysed in duplicate using liquid chromatography-tandem mass spectroscopy as previously described [21].

### Statistical analysis

Data are presented as means ± standard deviation (SD). Prior to analysis all data was assessed for normality using the Shapiro-Wilk test. To investigate the within-group and between-group effects of smoking we employed a mixed-design analysis of variance model [22]. For each dependent variable the model specified a main effect for treatment (pre- and post acute smoking) and a main effect for group (non-smokers and habitual smokers). If a significant condition x group interaction was observed, indicating that the effect of acute smoking differed between non-smokers and habitual smokers, within-group and between-group data were further analysed using post-hoc contrasts (Sidak adjusted). Data were also analysed with the change in nicotine levels and change in BRS as covariates. Pearson’s product-moment correlation coefficients were used to examine relationship between BRS and cerebral TFA metrics. Since multiple relationships were examined, p values were adjusted for multiple testing using the Holm-Bonferroni method. Statistical significance was set at p<0.05. All data were analysed using the statistical package SPSS (IBM SPSS statistics version 20, Surrey, UK).

## Results

All but one non-smoking participant completed the study. Participant characteristics are shown in Table 1. The two groups were comparable for age, gender and BMI but differed in their smoking habits. Habitual smokers, had an average 13 ± 5 pack years smoking history compared to 0 pack years in the non-smoking group. Consistent with these findings, cotinine was detected in the pre-smoking blood samples of habitual smokers only (Table 1).

**Table 1:** Demographic characteristics of the participating non-smokers and habitual smokers.

	Non-smokers (n=17)	Habitual smokers (n=15)
Age (years)	33 ± 12	37 ± 11
Male: Female ratio	10:7	8:7
Weight (kg)	77 ± 12	80 ± 13
Height (cm)	170 ± 10	172 ± 12
BMI (kg m^-2^)	26.8 ± 4.6	27.4 ± 4.2
Duration of smoking (pack years)	0 ± 0	13 ± 5**
Cotinine (ng mL^-1^)	0 ± 0	135 ± 82**

Values are means ± SD. BMI, body mass index. **p < 0.01 *vs.* non-smokers.

Results for nicotine concentrations pre- and post-acute smoking are summarised in Table 2. Habitual smokers had raised nicotine levels at baseline but neither group displayed any symptoms of unwellness. For habitual smokers and non-smokers alike, acute smoking led to a rise in nicotine, which was greater in the habitual smokers. Both groups had a similar increase in VAS after smoking.

**Table 2:** Baseline parameters in non-smokers and habitual smokers before (pre) and after (post) acute smoking.

	Non-smokers (n=17)		Habitual smokers (n=15)		p-values		
	Pre-smoking	Post-smoking	Pre-smoking	Post-smoking	Interaction	Treatment	Group
Nicotine (ng mL^-1^)	0.0 ± 0.0	4.5 ± 2.0**	4.1 ± 5.1^tt^	16.8 ± 7.9** ^tt^	<0.001	<0.001	<0.001
VAS (out of 10)	0.0 ± 0.0	6.0 ± 1.0	0.0 ± 0.0	6.0 ± 2.0	0.87	0.00	0.87
HR (beats min^-1^)	61 ± 7	69 ± 12	64 ± 10	72 ± 10	0.76	0.00	0.37
SAP (mmHg)	117 ± 16	121 ± 16	108 ± 9	111 ± 9	0.90	0.19	0.02
DAP (mmHg)	62 ± 10	67 ± 8	61 ± 10	66 ± 9	0.75	0.01	0.85
MAP (mmHg)	79 ± 13	84 ± 10	78 ± 9	83 ± 9	0.60	0.01	0.59
MCAv (cm s^-1^)	69 ± 14	69 ± 13	72 ± 9	73 ± 11	0.18	0.69	0.40
CVCi (cm s^-1^ mmHg)	0.89 ± 0.21	0.84 ± 0.23	0.93 ± 0.16	0.89 ± 0.17	0.76	0.04	0.50
P_ET_CO_2_ (mmHg)	39 ± 2	37 ± 2	38 ± 2	37 ± 3	0.06	0.00	0.39

Values are means ± SD. VAS, visual analogue scale; HR, heart rate; SAP, systolic arterial pressure; DAP, diastolic arterial pressure; MAP, mean arterial pressure; MCAv, mean cerebral blood flow velocity in the middle cerebral artery; CVCi, cerebrovascular conductance index; P_ET_CO_2_, partial pressure of end-tidal CO_2_. When an interaction was observed, symbols for post-hoc analysis were ^**^p < 0.01 *vs.* pre-smoking values and ^tt^p < 0.01 *vs.* non-smokers.

Table 2 also shows the cardiovascular, cerebrovascular and respiratory parameters pre- and post-acute smoking. There was no interaction for these parameters, indicating the response to acute smoking was similar in the two groups. Specifically, acute smoking evoked increases in HR, DAP and MAP, decreases in CVCi and P_ET_CO_2_, but no alteration in SAP or MCAv. With the exception of SAP, which was lower in habitual smokers, no group effects were detected. Adding the change in nicotine as a covariate exposed an interaction for P_ET_CO_2_ reflected by a greater post-smoking reduction in P_ET_CO_2_ in non-smokers (Table 3). Also, whilst the acute smoking-induced increase in HR remained, the effects of acute smoking on DAP, MAP, CVCi and were no longer significant (Table 3).

**Table 3:** Baseline parameters in non-smokers and habitual smokers before (pre) and after (post) acute smoking having controlled for nicotine as a covariate.

	Non-smokers (n=17)		Habitual smokers (n=15)		p-values		
	Pre-smoking	Post-smoking	Pre-smoking	Post-smoking	Interaction	Treatment	Group
HR (beats min^-1^)	61 ± 7	69 ± 12	64 ± 10	72 ± 10	0.41	0.04	0.39
SAP (mmHg)	117 ± 16	121 ± 16	108 ± 9	111 ± 9	0.78	0.44	0.09
DAP (mmHg)	62 ± 10	67 ± 8	61 ± 10	66 ± 9	0.87	0.51	0.96
MAP (mmHg)	79 ± 13	84 ± 10	78 ± 9	83 ± 9	0.97	0.46	0.73
MCAv (cm s^-1^)	69 ± 14	69 ± 13	72 ± 9	73 ± 11	0.15	0.31	0.50
CVCi (cm s^-1^ mmHg)	0.89 ± 0.21	0.84 ± 0.23	0.93 ± 0.16	0.89 ± 0.17	0.75	0.54	0.75
P_ET_CO_2_ (mmHg)	39 ± 2	37 ± 2**	38 ± 2	37 ± 3	0.02	0.07	0.26

Values are means ± SD. HR, heart rate; SAP, systolic arterial pressure; DAP, diastolic arterial pressure; MAP, mean arterial pressure; MCAv, mean cerebral blood flow velocity in the middle cerebral artery; CVCi, cerebrovascular conductance index; P_ET_CO_2_, partial pressure of end-tidal CO_2_. p-values for the interaction, treatment and group effects are presented with the change in nicotine as a covariate. ^**^p < 0.01 vs. pre-smoking values.

Cerebral TFA metrics pre- and post-acute smoking are shown in Table 4. There were no interaction or group effects for any of the measured parameters (MCAv power, MAP power, gain, n-gain, phase and coherence in all frequency ranges) after acute smoking. Acute smoking evoked alterations in MCAv power_HF_, MAP power_total_ and MAP power_VLF_. It also evoked reductions in gain_LF_, n-gain_LF ,_ coherence_LF_, and increases in phase_VLF_ and phase _LF_. Furthermore, the results were no different when the change in BRS was added to the GLM analysis (not tabulated).

**Table 4:** Transfer function analysis of spontaneous changes in arterial pressure and cerebral blood flow velocity in non-smokers and habitual smokers before (pre) and after (post) acute smoking.

	Non-smokers (n=17)		Habitual smokers (n=15)		p-values		
	Pre-smoking	Post-smoking	Pre-smoking	Post-smoking	Interaction	Condition	Group
MCAv_Total_ power (cm s ^-1^)^2^	14.24 ± 10.01	12.57 ± 6.96	15.23 ± 6.24	15.15 ± 9.38	0.22	0.74	0.29
MCAv_VLF_ power (cm s ^-1^)^2^	9.76 ± 8.32	7.49 ± 4.08	10.56 ± 6.01	10.06 ± 7.85	0.15	0.94	0.24
MCAv_LF_ power (cm s ^-1^)^2^	3.42 ± 2.46	3.26 ± 2.26	3.45 ± 1.32	2.37 ± 1.06	0.13)	0.07	0.51
MCAv_HF_ power (cm s ^-1^)^2^	0.99 ± 0.80	1.20 ± 0.84	1.00 ± 0.87	1.50 ± 1.08	0.37	0.04	0.59
MAP_Total_ power (mmHg^2^)	9.93 ± 4.41	11.45 ± 5.61	8.45 ± 4.10	12.02 ± 7.20	0.17	0.01	0.74
MAP_VLF_ power (mmHg^2^)	6.71 ± 3.94	7.36 ± 3.57	5.28 ± 2.99	7.91 ± 5.60	0.10	0.02	0.68
MAP_LF_ power (mmHg^2^)	2.59 ± 1.17	3.23 ± 2.47	2.53 ± 1.14	2.70 ± 1.03	0.47	0.17	0.55
MAP_HF_ power (mmHg^2^)	0.55 ± 0.45	0.80 ± 0.80	0.48 ± 0.57	0.64 ± 0.48	0.71	0.11	0.53
Gain_VLF_ (cm s^-1^ mmHg^-1^)	0.87 ± 0.36	0.84 ± 0.26	1.10 ± 0.30	0.97 ± 0.30	0.50	0.24	0.15
Gain_LF_ (cm s^-1^ mmHg^-1^)	1.04 ± 0.31	0.96 ± 0.27	1.19 ± 0.36	0.90 ± 0.23	0.11	0.01	0.64
Gain_HF_ (cm s^-1^ mmHg^-1^)	1.33 ± 0.42	1.27 ± 0.39	1.42 ± 0.45	1.46 ± 0.52	0.37	0.89	0.35
n-gain_VLF_ (% mmHg^-1^)	1.17 ± 0.34	1.20 ± 0.25	1.50± 0.47	1.33 ± 0.54	0.60	0.15	0.33
n-gain_LF_ (% mmHg-^1^)	1.49 ± 0.27	1.38 ± 0.32	1.69 ± 0.67	1.28 ± 0.39	0.07	0.01	0.77
n-gain_HF_ (% mmHg^-1^)	1.91 ± 0.51	1.83 ± 0.34	2.00 ± 0.64	1.98 ± 0.65	0.74	0.53	0.50
Phase_VLF_ (rad)	0.69 ± 0.53	1.07 ± 0.49	0.88 ± 0.45	1.05 ± 0.41	0.45	0.01	0.40
Phase_LF_ (rad)	0.51 ± 0.19	0.49 ± 0.17	0.56 ± 0.24	0.68 ± 0.21	0.07	0.04	0.06
Phase_HF_ (rad)	0.02 ± 0.18	0.07 ± 0.17	0.01 ± 0.17	0.09 ± 0.18	0.75	0.14	0.86
Coherence_VLF_ (AU)	0.46 ± 0.15	0.46 ± 0.14	0.54 ± 0.15	0.51 ± 0.15	0.57	0.70	0.13
Coherence_LF_ (AU)	0.69 ± 0.11	0.63 ± 0.15	0.69 ± 0.15	0.64 ± 0.17	0.86	0.04	0.97
Coherence_HF_ (AU)	0.76 ± 0.14	0.74 ± 0.16	0.75 ± 0.14	0.79 ± 0.09	0.33	0.65	0.66

Values are means ± SD. MCAv, mean cerebral blood flow velocity in the middle cerebral artery; MAP, mean arterial pressure; n-gain, normalized gain; VLF, very low frequency (0.02-0.07 Hz); LF, low frequency (0.07-0.2 Hz); HF, high frequency (0.20-0.35 Hz). All values are analysed with coherence. Unpaired t-test revealed no significant differences between pre-smoking values in non-smokers and habitual smokers. When an interaction was observed, symbols for post-hoc analysis were *p < 0.05 *vs.* pre-smoking values.

Cerebral TFA metric pre- and post smoking, having controlled for the change in nicotine levels are shown in Table 5. Adjusting for the change in nicotine as a covariate revealed interaction effects for n-gain_LF_ and phase_LF_ reflecting post-smoking reductions and increases in n-gain_LF_ and phase_LF_, respectively, in habitual smokers only. After this adjustment the post-smoking reduction in gain_LF_ and n-gain_LF_ were the only metrics showing treatment effects.

**Table 5:** Transfer function analysis of spontaneous changes in arterial pressure and cerebral blood flow velocity in non-smokers and habitual smokers before (pre) and after (post) acute smoking having controlled for nicotine as a covariate.

	Non-smokers (n=17)		Habitual smokers (n=15)		p-values		
	Pre-smoking	Post-smoking	Pre-smoking	Post-smoking	Interaction	Condition	Group
MCAv_Total_ power (cm s ^-1^)^2^	14.24 ± 10.01	12.57 ± 6.96	15.23 ± 6.24	15.15 ± 9.38	0.78	0.65	0.97
MCAv_VLF_ power (cm s ^-1^)^2^	9.76 ± 8.32	7.49 ± 4.08	10.56 ± 6.01	10.06 ± 7.85	0.34	0.93	0.84
MCAv_LF_ power (cm s ^-1^)^2^	3.42 ± 2.46	3.26 ± 2.26	3.45 ± 1.32	2.37 ± 1.06	0.10	0.23	0.74
MCAv_HF_ power (cm s ^-1^)^2^	0.99 ± 0.80	1.20 ± 0.84	1.00 ± 0.87	1.50 ± 1.08	0.75	0.49	0.31
MAP_Total_ power (mmHg^2^)	9.93 ± 4.41	11.45 ± 5.61	8.45 ± 4.10	12.02 ± 7.20	0.80	0.87	0.26
MAP_VLF_ power (mmHg^2^)	6.71 ± 3.94	7.36 ± 3.57	5.28 ± 2.99	7.91 ± 5.60	0.71	0.88	0.04
MAP_LF_ power (mmHg^2^)	2.59 ± 1.17	3.23 ± 2.47	2.53 ± 1.14	2.70 ± 1.03	0.47	0.57	0.25
MAP_HF_ power (mmHg^2^)	0.55 ± 0.45	0.80 ± 0.80	0.48 ± 0.57	0.64 ± 0.48	0.46	0.82	0.51
Gain_VLF_ (cm s^-1^ mmHg^-1^)	0.87 ± 0.36	0.84 ± 0.26	1.10 ± 0.30	0.97 ± 0.30	0.90	0.80	0.12
Gain_LF_ (cm s^-1^ mmHg^-1^)	1.04 ± 0.31	0.96 ± 0.27	1.19 ± 0.36	0.90 ± 0.23	0.07	0.03	0.54
Gain_HF_ (cm s^-1^ mmHg^-1^)	1.33 ± 0.42	1.27 ± 0.39	1.42 ± 0.45	1.46 ± 0.52	0.25	0.40	0.19
n-gain_VLF_ (% mmHg^-1^)	1.17 ± 0.34	1.20 ± 0.25	1.50± 0.47	1.33 ± 0.54	0.98	0.89	0.24
n-gain_LF_ (% mmHg-^1^)	1.49 ± 0.27	1.38 ± 0.32	1.69 ± 0.67	1.28 ± 0.39*	0.05	0.02	0.53
n-gain_HF_ (% mmHg^-1^)	1.91 ± 0.51	1.83 ± 0.34	2.00 ± 0.64	1.98 ± 0.65	0.77	0.88	0.27
Phase_VLF_ (rad)	0.69 ± 0.53	1.07 ± 0.49	0.88 ± 0.45	1.05 ± 0.41	0.70	0.23	0.08
Phase_LF_ (rad)	0.51 ± 0.19	0.49 ± 0.17	0.56 ± 0.24	0.68 ± 0.21*	0.02	0.05	0.04
Phase_HF_ (rad)	0.02 ± 0.18	0.07 ± 0.17	0.01 ± 0.17	0.09 ± 0.18	0.10	0.74	0.83
Coherence_VLF_ (AU)	0.46 ± 0.15	0.46 ± 0.14	0.54 ± 0.15	0.51 ± 0.15	0.51	0.80	0.94
Coherence_LF_ (AU)	0.69 ± 0.11	0.63 ± 0.15	0.69 ± 0.15	0.64 ± 0.17	0.75	0.29	0.48
Coherence_HF_ (AU)	0.76 ± 0.14	0.74 ± 0.16	0.75 ± 0.14	0.79 ± 0.09	0.61	0.85	0.71

Values are means ± SD. MCAv, mean cerebral blood flow velocity in the middle cerebral artery; MAP, mean arterial pressure; n-gain, normalized gain; VLF, very low frequency (0.02-0.07 Hz); LF, low frequency (0.07-0.2 Hz); HF, high frequency (0.20-0.35 Hz). All values are analysed with coherence. Unpaired t-test revealed no significant differences between pre-smoking values in non-smokers and habitual smokers. In this table p-values for the interaction, treatment and group effects are presented with the change in nicotine as a covariate. When an interaction was observed, symbols for post-hoc analysis were *p < 0.05 *vs.* pre-smoking values.

**Figure 1:**
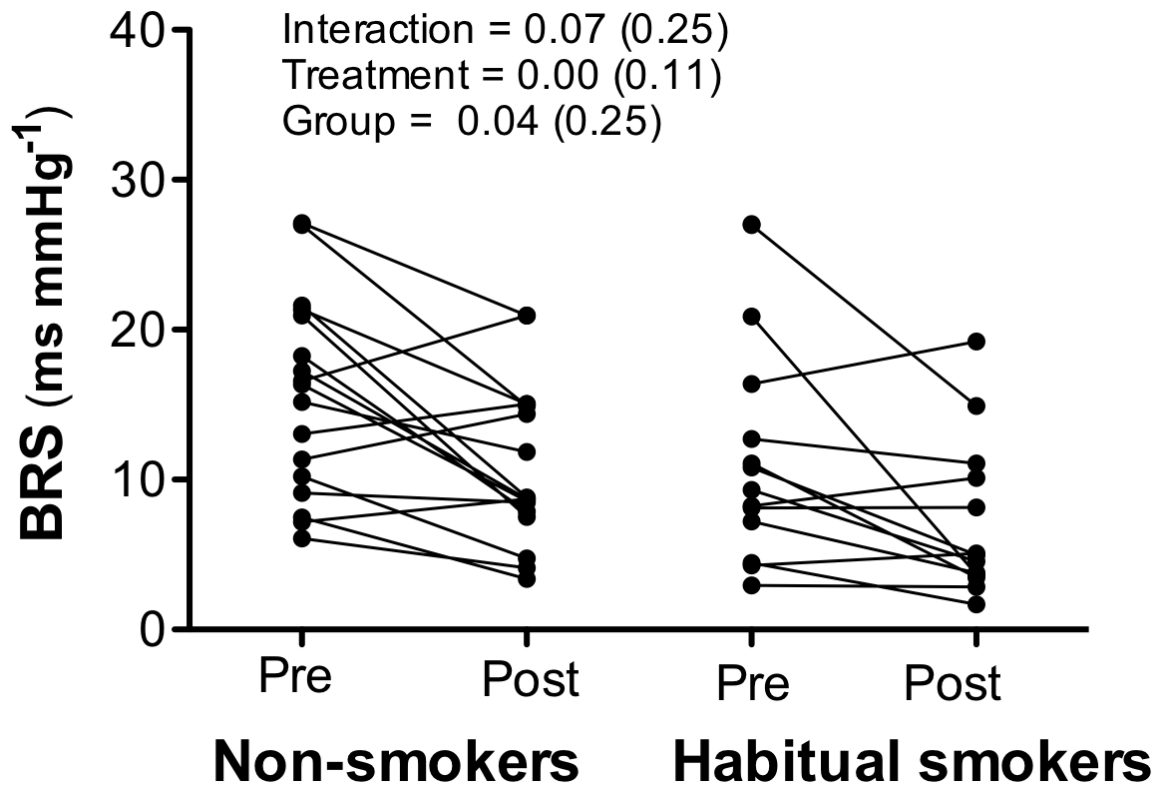
The effects of acute and chronic smoking on baroreflex sensitivity (BRS). All non-smoking participants met the ≥0.5 coherence criteria for computation of BRS. Two habitual smokers were not included in BRS analysis, as their post-smoking BRS did not meet this coherence criteria.

TFA of BRS pre- and post-smoking is shown in Figure 1. There were no interaction effects, irrespective of whether or not the data were adjusted for the change in nicotine. Before, but not after, adjusting for the change in nicotine as a covariate both groups showed a post-smoking reduction in BRS, which was greater in habitual smokers.

**Figure 2:**
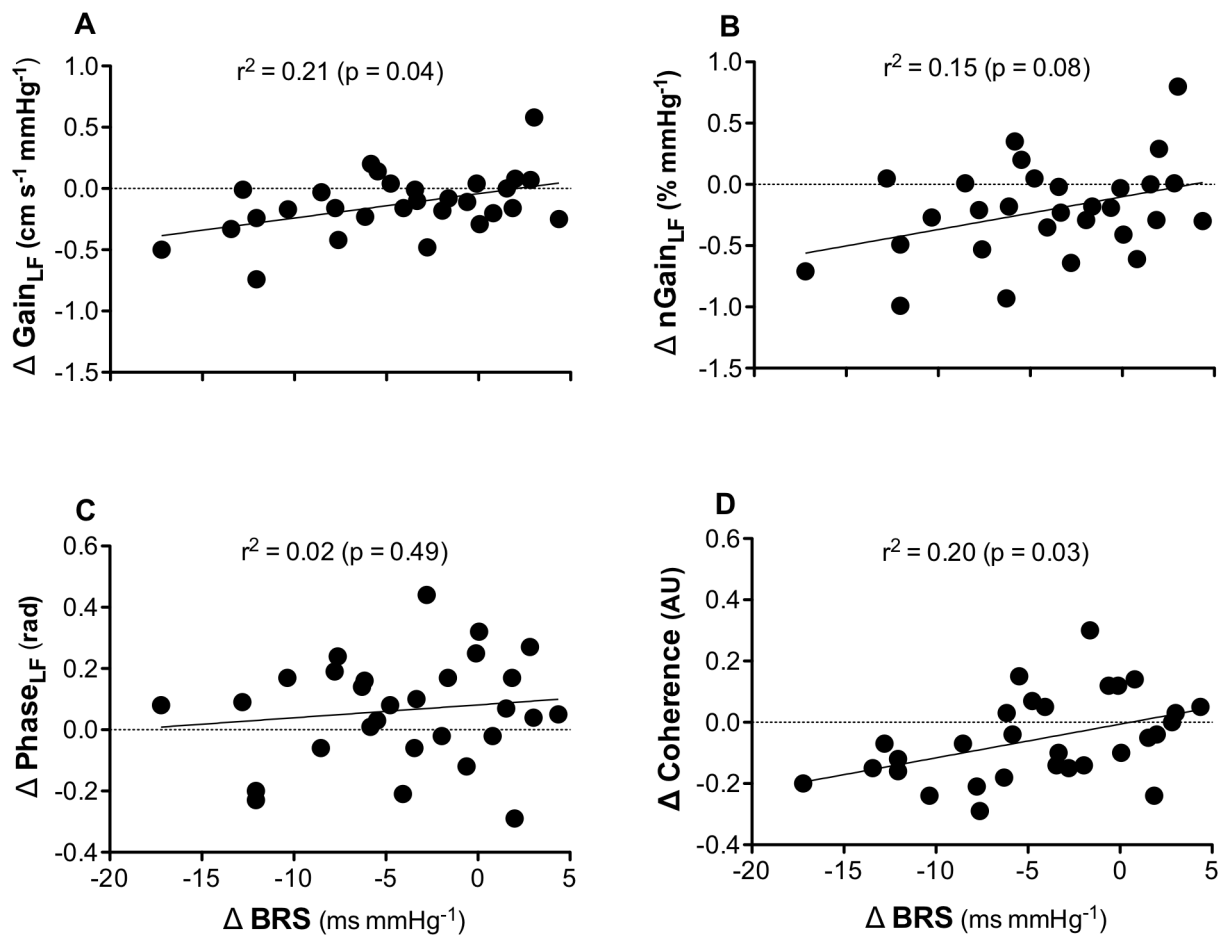
Correlations between the change in BRS and changes in cerebral transfer function metrics after acute smoking. There were significant correlations between the change in BRS and: i) gain (panel A) and, ii) coherence (panel D) in the low frequency. There was no correlation between the change in BRS and i) normalised gain (n-gain, panel B) and ii) phase (panel C) in the low frequency.

Regression analysis (Figure 2) showed that 21% of the reduction in gain_LF_ and 20% of the reduction in coherence_LF_ were explained by a reduction in BRS. All these relationships were significant, irrespective of whether or not nicotine was added as a covariate. A relationship was not detected between n-gain_LF_ and BRS, or phase_LF_ and BRS.

## Discussion

This study assessed the effects of acute and habitual smoking on cerebral pressure-flow dynamics, and cardiac BRS. Cerebral TFA showed that acute smoking reduced gain, reduced coherence, and increased phase in non-smokers and habitual smokers. We interpret these findings as indirect evidence that acute smoking *improved* the pressure buffering capacity of the cerebral circulation, possibly due to enhanced CA. However, gain, phase and coherence were similar between non-smokers and habitual smokers before acute smoking, which suggests that chronic smoking did not alter intracranial pressure-flow dynamics. Furthermore, regression analysis revealed a weak correlation between reductions in gain and coherence, and the reduction in BRS.

### Effect of acute smoking on cardiovascular and cerebrovascular parameters

Our observations that acute cigarette smoking increases HR, DAP and MAP are consistent with others [3,4,23]. Although this study was not designed to establish the mechanism/s for these changes, sympathetic nerve activation may be involved [23,24]. The increase in HR, persisted when the change in nicotine was added as a covariate, suggesting constituents of a cigarette, besides nicotine may augment sympathetic activity. In this regard, acute smoking is also associated with an increase in oxidative stress and consequent reduction in nitric oxide (NO) [25] which have tonic excitatory effects on central sympathetic transmission [26]. However, the observation that the acute smoking-induced increases in DAP and MAP were not significant when nicotine was added as a covariate, suggests nicotine was a confounding factor.

Our findings of an acute smoking-induced reduction in spontaneous BRS is also in keeping with others [3,4]. This may be related to a reduction in arterial compliance since acute smoking reduces compliance in systemic conduit vessels, including the carotid artery [27,28]. We also found that acute smoking had no effect on resting MCAv whereas others have found that MCAv is increased [8] and decreased [29] after acute smoking. However, the effects of smoking on resting MCAv are known to be contradictory [29] possibly due to the lack of comparability in the smoking stimulus.

Acute smoking evoked reductions in gain_LF_, and coherance_LF_, and an increase in phase_LF_, which suggests it dampened the CBF response to alterations in pressure, reduced the degree to which CBF and pressure are linearly related, and increased the phase lag between changes in CBF and pressure, respectively. One explanation for these changes is that they reflect an improvement in cerebral autoregulation (CA) possibly due to reductions in P_ET_CO_2_ following acute smoking. That said, the magnitude of this reduction is small, hence unlikely to have any appreciable effects on MCA diameter. Serrador et al. [30] combined transcranial Doppler ultrasound and magnetic resonance imaging and demonstrated that MCA diameter is unchanged during moderate reductions in P_ET_CO_2_ (i.e. down to 24 mmHg). Another explanation is that the reduction in gain and increase in phase after acute smoking, are attributed, at least in part, to changes in steady-state properties of the cerebrovascular bed. Zhang et al. [31] observed a reduction in gain and increase in phase between spontaneous BP and CBF velocity after phenylephrine infusion and this led to an increase in cerebrovascular resistance and decrease compliance in their healthy volunteers. We observed a reduction in CVCi after acute smoking which suggests that acute smoking increased cerebral vascular resistance and potentially reduced compliance. Such changes could contribute to the reductions in gain and coherence, and an increase in phase after acute smoking. Regardless of the mechanisms, contrary to our original hypothesis our findings indicate the cerebral circulation is *less* pressure-passive after acute smoking.

The notion that acute smoking improves dynamic CA contravenes previous studies showing that acute smoking reduces hypercapnic cerebral vascular reactivity [8,9]. However, previous studies assessed cerebral vascular response to CO_2_ in a limited hypercapnic range and did not include a hypocapnic stimulus. Furthermore, a reduction in CO_2_ reactivity does not necessarily mean a reduction in the cerebrovascular response to alteration in pressure. For example, the release of endothelial-derived factors plays a predominant role in the cerebral vasodilatory response to hypercapnia [32], whereas myogenic factors play a predominant role in alteration in CBF in response to changes in intravascular pressure [33].

### Effect of chronic smoking on cardiovascular and cerebrovascular parameters

Apart from a lower BRS in habitual smokers, there were no between-group differences in HR, BP, MCAv, or cerebral TFA metrics in the baseline state. These findings were surprising given that chronic smoking causes hypertension and can reduce CBF [7,34]. Also, chronic smoking has deleterious effects on the structure of the cerebral vasculature [35–37] which would be expected to impair its ability to respond to BP alterations. The absence of cardiovascular and cerebrovascular impairment in our habitual smokers may relate to their relatively light smoking habit (13 mean pack years) and relative youth (mean of 37 years). Participants in previous studies showing that chronic smoking reduces CBF had a more long-standing smoking habit (≥24 pack years) and were older (≥55 years) [7,34]

### The dynamic relationship between pressure and flow and baroreflex sensitivity

We found weak correlations between an increase in gain_LF_ (and coherence_LF_) and a reduction in BRS. These findings are mechanistically difficult to explain. It is tempting to suggest that the reduction in BRS mediated a compensatory improvement in cerebrovascular regulation. However against this suggestion, these reductions in gain and coherence remained after controlling for BRS as a covariate. An alternative possibility is that smoking directly influenced calcium signalling in cerebrovascular myocytes. Several recent studies have demonstrated that smoking activates Rho-kinase in forearm blood vessels, possibly via a decrease in NO and/or increase in Endothelin-I (a vasoconstrictor) [38,39]. Rho-kinase promotes vascular smooth muscle (VSM) contraction, secondary to the inhibition of myosin light chain phosphatase and an increase in calcium sensitivity [40,41]. Assuming smoking also increases in Rho-kinase in cerebral vessels, Rho-kinase activation may augment the sensitivity of cerebral VSM cells to pressure fluctuations.

### Limitations

Several study limitations warrant consideration. First, MCA diameter may have changed after acute smoking, thereby invalidating MCAv as a surrogate index of CBF. Iida and colleagues [42] demonstrated, in rats, that acute smoking led to biphasic changes in pial artery diameter (30 s contraction followed by relaxation that peaked at ~ 5-10 min and returned to baseline in an hour). Even so, the majority of cerebral TFA metrics (phase and coherence) are independent of MCA diameter changes. Second, despite training, our non-smokers extracted less nicotine from a single cigarette than habitual smokers. It is possible that non-smokers subtlely adapted their smoking style to ease the fast-acting and unpleasant effects of smoking (e.g. dizziness). However, adjusting for the change in nicotine levels as a covariate accounted for this potential confounder. Third, by design we selected a habitual smoking population, that was free from known vascular and respiratory co-morbidities. The byproduct of this screening process was that our habitual smokers had a relatively mild smoking habit and were relatively young, which may account for the absence of any between-group differences in baseline haemodynamics and cerebral TFA metrics.

### Perspectives

Assuming that the cerebral pressure-flow relationship reflects an improvement in CA, the present findings would suggest that acute smoking *enhances* the brain’s ability to respond to BP alterations. This finding would appear to have teleological benefit. By buffering the brain from increases and decreases in BP it protects the delicate cerebral vessels from barotrauma, thereby mitigating end-organ damage and stroke risk.

Our relatively young smokers with a mild-moderate smoking habit did not exhibit any alterations in baseline cerebral TFA, which would have suggested impaired CA, despite a reduction in BRS. This shifts our assumption that the damaging effects of cigarette smoking on systemic and cerebral vasculature occur in parallel. However future studies investigating the cerebrovascular effects of cigarette smoking in habitual smokers with a greater smoking habit, and controlling for age, are warranted.
